# Effect of translational shear on interfacial structure in the viscous fingering instability

**DOI:** 10.1126/sciadv.aeb2907

**Published:** 2026-04-03

**Authors:** Zhaoning Liu, Samar Alqatari, Thomas E. Videbæk, Sidney R. Nagel

**Affiliations:** ^1^ Department of Physics and The James Franck and Enrico Fermi Institutes, University of Chicago, Chicago, IL 60637, USA.; ^2^ Martin A. Fisher School of Physics, Brandeis University, Waltham, MA 02453, USA.

## Abstract

We introduce applied shear as a method to control viscous fingering by smoothing the interface between miscible fluids. In the viscous fingering instability, a less viscous fluid displaces a more viscous one through the formation of fingers. The instability, which requires a confined geometry, is often studied in the thin gap of a quasi–two-dimensional Hele-Shaw cell. When the two fluids are miscible, the structures that form in the dimension traversing the gap are important for determining the instability onset. We demonstrate with experiments and simulations that oscillatory translational shear of the confining plates changes the gap-averaged viscosity profile so that it becomes less abrupt at the fingertips. Increasing the amplitude or velocity of the shear delays the instability onset and decreases the finger growth rate. Shear can thus be used to stabilize a pair of miscible fluids against fingering. The results show a direct correlation between a smoother viscosity profile and delayed instability.

## Teaser

Oscillatory shear smooths fluid interfaces, stabilizing viscous fingering in miscible fluids.

## INTRODUCTION

Viscous fingering is a prototypical example of pattern formation. It occurs at the interface between two fluids when the fluid with lower viscosity displaces the other in a confined geometry such as a thin gap between two surfaces (*1*). At the instability onset, the interfluid interface develops lateral undulations that develop into fingers as the less-viscous fluid protrudes into the fluid that fills the rest of the gap. The conditions for this to occur generally depend on the viscosities of the two fluids, the interfacial tension, and the local velocity of the interface (*1*–*5*).

**Fig. 1.**
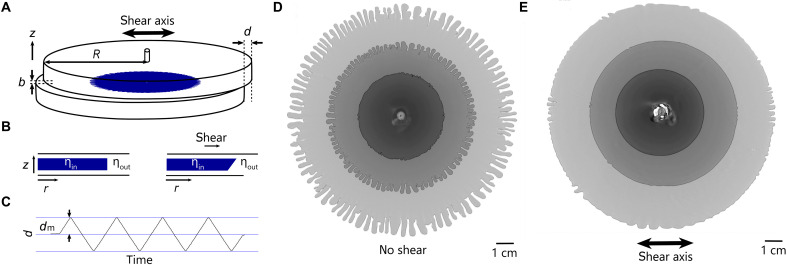
Experiment: Viscous fingering under oscillatory shear. (**A**) Schematic for a radial Hele-Shaw cell where the top plate is displaced from the bottom one by an amount d. For measurements without shear, we align the plates with d=0. The gap between the plates extends from z=0 to z=b. b is much smaller than the radius of the plates, R. Fluid is injected from an inlet at the center of the top plate. (**B**) Schematic of the effect of shear on a stationary vertical interface shown on left; after shear (right), the interface is tilted. (**C**) Measured displacement, d, between the centers of the two plates is varied with a triangular waveform with amplitude $d_{m}$ and velocity $\pm V_{s}$. (**D** and **E**) Comparison of interfaces from experiments (D) with no shear and (E) with shear with $d_{m}=3\;\text{mm}$ and $V_{s}=5.6\;\text{mm}/s$. Darker images show earlier times. Images with the same gray scale in (D) and (E) are taken at the same radius. The smallest (i.e., darkest) image in (D) is taken when fingers first appear in the absence of shear. In (E) at the same radius, the interface shows no finger formation. Fingers only begin to form in (E) at the second image. For both cases, b=305 μm, Q=133 μL/s, $\eta_{\text{out}}=218\pm 5\;\text{mPa}\cdot s$, and $\eta_{\text{in}}=35\pm 2\;\text{mPa}\cdot s$.

As the interfacial tension drops, the fingers become thinner but do not become arbitrarily narrow, remaining finite even in the limit of miscible fluids where the interfacial tension nearly vanishes (*6*–*8*). Paradoxically, in that case, although the stabilizing effect of the interfacial tension is removed, a regime occurs where the fluids become stable against lateral fingering; although the invading fluid is less viscous, fingers may not form as they would for pairs of immiscible fluids. In that situation, for fingering to occur, the interface between the fluids must be sufficiently blunt in the direction spanning the gap [i.e., in the z direction as illustrated in Fig. 1 (A and B)] (*9*–*13*).

The interface bluntness is controlled by the ratio of the inner to outer viscosity, $\eta_{\text{in}}/\eta_{\text{out}}$ (*9*–*14*). In addition, if the injection rate is sufficiently low, then the interface between miscible fluids can become smeared out due to interfluid diffusion (*15*–*18*); this leads to a delayed instability onset and a slower finger growth rate (*19*). Thus, both the geometry (i.e., the bluntness) and the diffusion independently alter the profile of the average viscosity near the finger tip. This raises issues of how sharp must the interface at the tip be in order for the fingering instability to occur and how does the variation of the gap-averaged viscosity near the tip control the onset and evolution of fingering.

A common experimental platform for studying viscous fingering is in a radial Hele-Shaw cell, illustrated in Fig. 1A, where two smooth horizontal glass plates of radius R are separated by a gap of width b with b≪R (*1*, *4*, *20*–*24*). The gap is first filled with the fluid of viscosity $\eta_{\text{out}}$, and then a lower viscosity fluid with viscosity $\eta_{\text{in}}$ is injected through a small hole in the center of one of the plates.

For miscible fluids, the displacing fluid forms a thin tongue in the gap protruding between two layers of the displaced fluid. As $\eta_{\text{in}}/\eta_{\text{out}}\to 1$, where the two fluids would have identical physical properties, the flow approaches a parabolic (Poiseuille) profile in the z direction traversing the gap so that the gap-averaged viscosity profile, $\langle \eta \left(r\right)\rangle \equiv \frac{1}{b}\int_{0}^{b}\;\eta \left(z,r\right)\mathrm{dz}$, gently increases as a function of radius, r (*25*, *26*). As $\eta_{\text{in}}/\eta_{\text{out}}$ decreases, 〈η(r)〉 at the interface between the inner and outer fluids becomes more blunt at the tip of the invading fluid (*9*, *11*). The quasi–two-dimensional lateral pattern becomes unstable to fingering for $\eta_{\text{in}}/\eta_{\text{out}}≲0.3$. This transition was associated with shock formation in 〈η(r)〉 (*9*, *10*). So far, it has not been possible to isolate the specific role of interface shape from other physical properties such as mobility, viscosity ratio, or diffusion. The theoretical analysis of the gap profile (*27*–*29*) includes a recent study that corroborated that a discontinuity in 〈η(r)〉 at the interface tip is required for fingering onset (*13*). It has remained unclear whether the onset can be continuously delayed by progressively smoothing the interface.

Here, we use translational oscillatory shear between the parallel plates of the Hele-Shaw cell to perturb the interface actively. This allows the shape of the interface to be altered independently from either the viscosity ratio or diffusion and directly tests whether the interface shape is indeed the critical driver of the instability. This experiment quantitatively correlates the smoothness at the leading edge of the fluid interface to the instability.

If the two fluids were initially stationary with the tip of their interface perfectly vertical, then shearing the top plate would tilt the interface as shown in the schematic in Fig. 1B. In this simplified view with no shear, the gap-averaged viscosity would have a discontinuous jump at the interface. However, when shear is applied, the tilting of the interface causes the gap-averaged viscosity to increase smoothly from the inner to the outer fluid. When the fluids are not at rest, the contour of the interfluid interface will depend on the shear-displacement amplitude, $d_{m}$, and the relative velocity of shear, $\pm V_{s}$, with respect to the advancing interface, U, which is proportional to the fluid injection rate, Q. Thus, shear can present an independent way to alter d〈η(r)〉/dr.

Because the cell is circular and the shear is along one chosen axis, the direction of shear relative to finger propagation varies around the pattern. Thus, we observe the effects of shear in two orthogonal directions. Here, we focus on fingers growing in the parallel direction where shear delays the instability onset and lowers the growth rate of fingers once formed. The effect of shear on fingers in the perpendicular direction will be presented in a later paper.

## RESULTS

### Experimental platform

Our Hele-Shaw cell consists of two large, flat, circular glass plates with a radius R=14 cm as illustrated in Fig. 1A. The gap spacing between the plates was kept uniform at b=305 μm by using six spacers of equal height placed around the cell edge. The top plate is maintained in contact with the washers by weights equally placed around the perimeter. The fluids are injected through a hole in the center of the top plate. Fingering patterns are imaged from below the plates.

During the fluid injection, the plates are cyclically sheared with respect to each other along one axis with a triangular waveform to a constant maximum amplitude, $d_{m}$, as shown in Fig. 1C. In different experiments, we varied $d_{m}$ and shear speed, $V_{s}$: $0.3\;\text{mm}<d_{m}<6\;\text{mm}$ and $1.4\;\text{mm}/s<V_{s}<28\;\text{mm}/s$.

Both the inner and outer fluids are mixtures of glycerol and water whose viscosities are $\eta_{\text{in}}=35\pm 2\;\text{mPa}\cdot s$ and $\eta_{\text{out}}=218\pm 5\;\text{mPa}\cdot s$, respectively. The inner fluid is dyed, so that the concentration profile of the inner fluid, C(r), can be measured from the transmitted light intensity as discussed in Materials and Methods (*11*, *19*). Initially, the gap between the plates is fully filled with the more viscous fluid. The less viscous displacing fluid is then injected using a syringe pump at a constant volume rate, Q, with values between 67 μl/s≤Q≤533 μl/s. As the interface propagates radially outward, its speed decreases as a function of radius: U=Q/(2br). We only tracked the interface before U decreases to 0.7 mm/s, which happens beyond the instability onset. This ensures that the Péclet number Pe≡Ub/D>1750, so that there is a well-defined interfluid interface (*19*), such that $\langle \eta \left(r\right)\rangle =\left(\eta_{\text{in}}-\eta_{\text{out}}\right)\cdot C\left(r\right)+\eta_{\text{out}}$. The effective interfluid diffusivity, D, is estimated to be 1.21 × 10^−10^ m^2^/s using Fickian diffusion of glycerol-water mixtures. Details of the experiment are given in Materials and Methods.

Our experiments investigate how the application of shear between the confining plates changes the onset radius of the fingering instability, $R_{\text{on}}$, and the growth rate of the fingers once they are formed, Γ. We measure the concentration profile of the inner fluid, C(r), and correlate its shape with $R_{\text{on}}$. We focus on fingers growing in the direction parallel to the shear axis.

### Effect of shear on onset radius and finger growth rate

Figure 1D shows three superimposed images from a conventional experiment without shear between the plates. The darkest image at the center, taken at the earliest-time, shows fingers just becoming visible; the lighter patterns, taken at later times, show well-developed fingers emerging in all directions. Two relevant features of these patterns are the onset of finger growth at a radius, $R_{\text{on}}$, which is set (in both miscible and immiscible pairs of fluids) by the viscosity ratio, $\eta_{\text{in}}/\eta_{\text{out}}$ (*30*), and a wavelength characterizing the finger widths.

Figure 1E shows the corresponding images taken while the plates were cyclically sheared during fluid injection. The shear is along the axis of the double-headed arrow. Aside from the shear, the two experiments had the same fluids, gap spacing, and injection rate. In Fig. 1E, the innermost pattern is smooth with no indication of incipient finger growth. This should be compared to the image with the same gray scale in Fig. 1D taken at the same radius where fingers had already formed in the absence of shear. Fingers only become visible at larger radii as seen in the image with a radius ~1.6 times larger than the first. Shear delays the onset of fingering substantially.

**Fig. 2.**
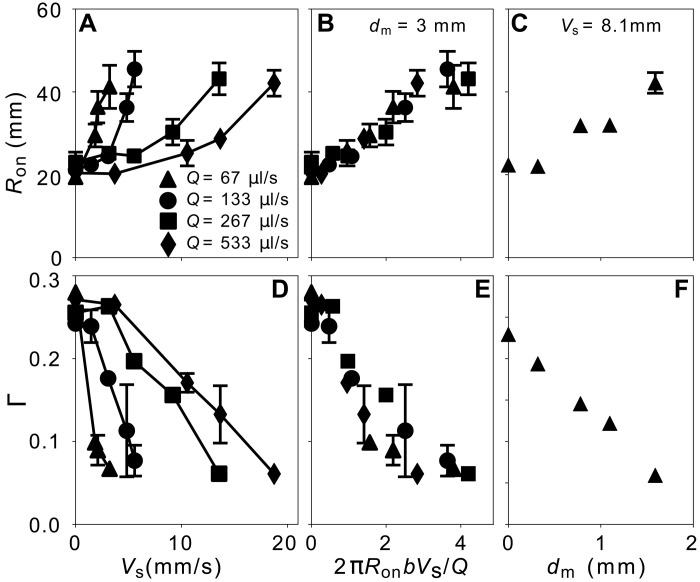
Effect of shear on instability onset and growth for fingers parallel to the shear axis. (**A** to **C**) show data for onset radius and (**D** to **F**) show data for growth rate. (A and D) Onset radius, $R_{\text{on}}$, and growth rate, Γ, versus shear speed $V_{s}$. (B and E) Same data as in (A) and (D), but $V_{s}$ is normalized to be dimensionless by the interface propagation speed at the onset radius, $Q/\left(2\pi R_{\text{on}}b\right)$. The curves collapse for the different injection rates Q. (C and F) $R_{on}$ and Γ versus $d_{m}$. All symbols share the same legend as (A). For all data shown here, the error bars, unless explicitly shown, are comparable to or smaller than the size of the symbols. The solid and dashed lines are guides to the eye.

Figure 2 quantifies how increasing the shear speed, $V_{s}$, or shear amplitude, $d_{m}$, delays the instability onset. As shown in Fig. 2A, $R_{\text{on}}$ increases with increasing $V_{s}$, while $d_{m}$ is held constant. This delay persists across different injection rates, Q. Normalizing the shear speed by the interface propagation speed at the onset radius, $Q/2\pi R_{\text{on}}b$, scales the data onto a single curve as shown in Fig. 2B. Figure 2C shows that increasing the shear amplitude $d_{m}$ while holding $V_{s}$ and Q constant also increases $R_{\text{on}}$.

Once the fingers have formed, shear also reduces the finger growth rate, $\Gamma \equiv {\mathrm{dR}}_{f}/{\mathrm{dR}}_{\text{tip}}$, where $R_{f}$ is the finger length and $R_{\text{tip}}$ is the outer extent of the finger. Similar to the case without shear (*12*), soon after onset, driven by pressure gradients in the bulk of the fluids (*31*), $R_{f}$ varies linearly with $R_{\text{tip}}$, as shown in fig. S10. Figure 1 (D to F) shows Γ just after onset, before $R_{\text{tip}}-R_{\text{on}}$ reaches 10 mm. Γ decreases with increasing $V_{s}$ as shown in Fig. 1D. The data for different injection rates collapse onto a single curve when $R_{\text{on}}$ and Γ are each plotted in Fig. 1 (B and E), respectively, versus the dimensionless variable $2\pi R_{\text{on}}{\mathrm{bV}}_{s}/Q$. (As noted, this variable compares the velocity of the shear to the velocity of injected fluid at the onset radius. However, the experimentally controlled variable in our experiment is $V_{s}/Q$.) Likewise, increasing $d_{m}$ at fixed $V_{s}$ and Q also suppresses Γ as shown in Fig. 2F. These results demonstrate that increasing either shear amplitude or speed increases the radius of onset, $R_{\text{on}}$, and also slows down the subsequent finger-growth dynamics, Γ.

### Transverse structure across the gap

We now investigate the correlation between the interface profile in the z direction across the gap and the onset of the fingering. To do this, we measure the gap-averaged inner fluid concentration profile C(r) along the radial direction r from the center of the inner fluid to the interface along a finger, as described in Materials and Methods.

**Fig. 3.**
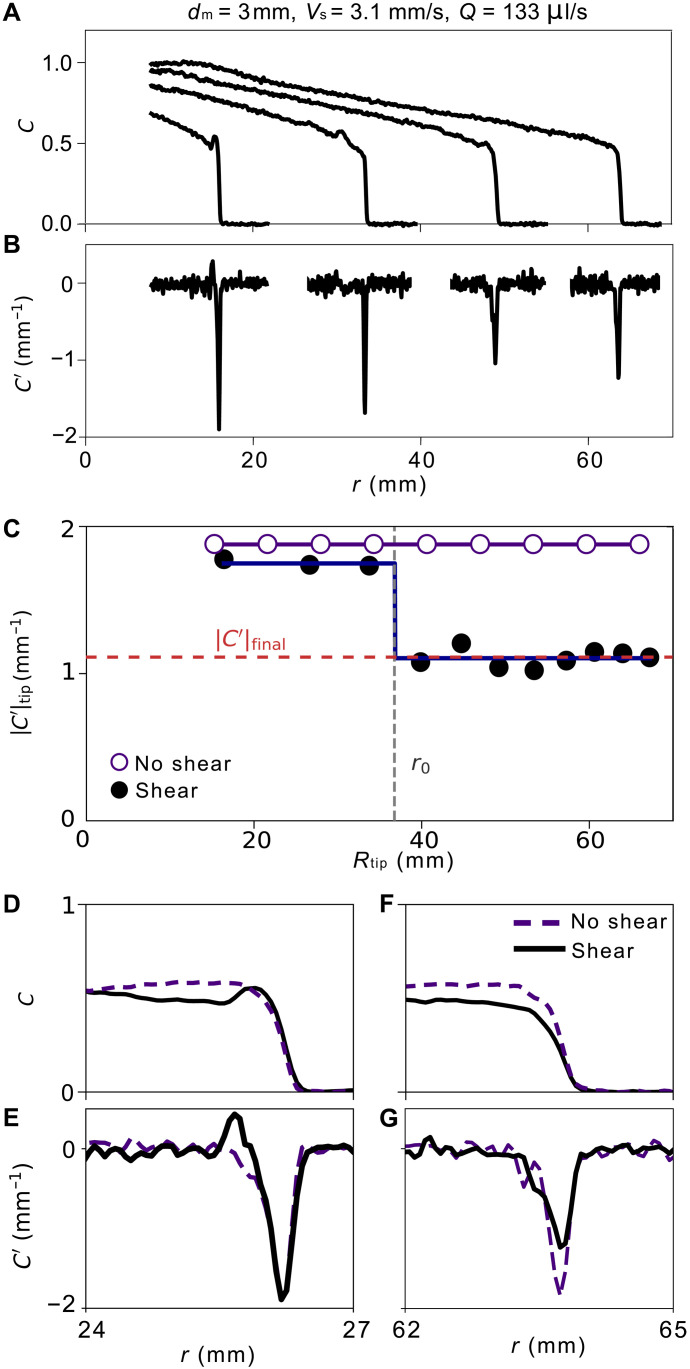
Effect of shear on the interface concentration profile. (**A**) Selected profiles of inner fluid concentration, C(r), taken at four positions, from left to right, $R_{\text{tip}}\approx 16\;\text{mm},33\;\text{mm},49\;\text{mm},\text{and}\;64\;\text{mm}$. (**B**) Radial derivative of the concentration profile, $C^{\prime }\left(r\right)$, measured near the finger tip at the same positions as in (A). (**C**) ${|C^{\prime }|}_{\text{tip}}$ versus the position of the tip $R_{\text{tip}}$. For the case with shear, the fit is to the step function described in the text. Without shear, ${|C^{\prime }|}_{\text{tip}}$ is approximately constant. (**D** and **E**) Concentration profile C(r) and its radial derivative $C^{\prime }\left(r\right)$ at $R_{\text{tip}}\approx 26\;\text{mm}<r_{0}\approx 37\;\text{mm}$. Both profiles show that the interface tip exhibits comparable bluntness with and without shear for $r<r_{0}$ . (**F** and **G**) At $R_{\text{tip}}\approx 64\;\text{mm}>r_{0}$, the interface tip under shear becomes noticeably smoother than the one without shear. (G) Magnitude of $C^{\prime }\left(r\right)$ at the tip with shear decreases by nearly one-third of that without shear.

Figure 3A shows an example of interface evolution under shear, which is tracked by measuring the concentration profile during the course of an experiment. To measure the smoothness or bluntness of the interface, we take the derivative of that concentration profile $C^{\prime }\left(r\right)\equiv \mathrm{dC}\left(r\right)/\mathrm{dr}$, as shown in Fig. 3B, and find that $C^{\prime }\left(r\right)$ dips abruptly at the end of a finger. The maximum of its absolute value, ${|C^{\prime }|}_{\text{tip}}$, reflects how blunt the finger is at its tip. The peaks in $|C^{\prime }\left(r\right)|$ decrease as the interface expands while being sheared. The corresponding evolution of the concentration profile and its radial derivative $C^{\prime }\left(r\right)$ without shear is shown in the fig. S6.

Figure 1C shows that when there is shear, ${|C^{\prime }|}_{\text{tip}}$ remains constant until the interface reaches $r_{0}\approx 37\;\text{mm}$, at which point it rapidly drops to a lower plateau value ${|C^{\prime }|}_{\text{tip}}={|C^{\prime }|}_{\text{final}}$. This behavior defines a length $r_{0}$, the smoothing radius, where shear begins to modify the interface. We quantify this transition with a step function: ${|C^{\prime }|}_{\text{tip}}={|C^{\prime }|}_{\text{final}}+C^{\prime }\Theta \left(r_{0}-r\right)$, where Θ(…) is the Heaviside step function. When there is no shear, ${|C^{\prime }|}_{\text{tip}}$ remains constant as the inner fluid expands. Additional examples for varying shear speeds and amplitudes are provided in fig. S7. The step-like behavior is representative of what we see across all our experiments. However, we cannot resolve the rapidity of the decrease with our current resolution.

Figure 3 (D and F) shows the concentration profiles C(r) near the tip for the case with shear compared to the case when there is no shear. When $R_{\text{tip}}<r_{0}$, the profile shapes are nearly the same, whereas when $R_{\text{tip}}>r_{0}$, the profile for the sheared case is smoother. Explicitly comparing the derivatives $C^{\prime }\left(r\right)$, shown in Fig. 1 (E and G), illustrate the change in interfacial bluntness across $r_{0}$, showing that shear decreases the bluntness of the interface tip.

**Fig. 4.**
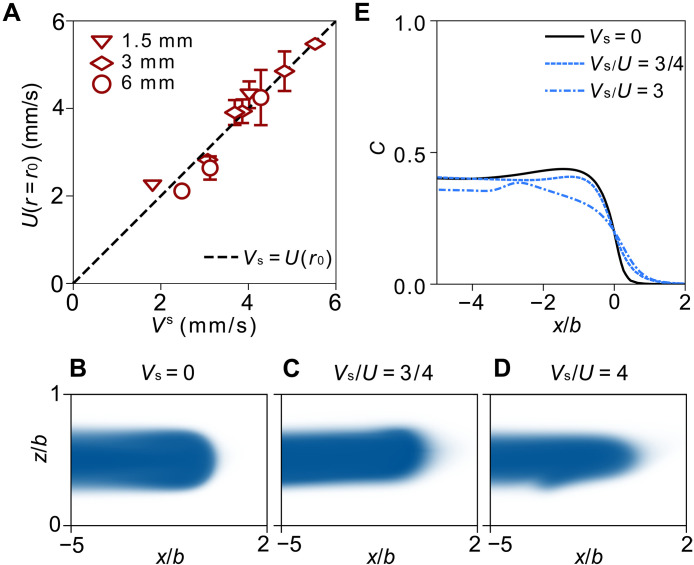
Competition between shear speed and interface velocity. (**A**) The experimentally measured interface speed at $r=r_{0}$, ,$U\left(r=r_{0}\right)$ versus the shear speed, $V_{s}$. The dashed line, with slope one, shows that $r_{0}$ is determined by when $U\left(r=r_{0}\right)=V_{s}$. (**B** to **D**) Two-dimensional COMSOL simulation results showing the shape of the inner fluid (blue) around its tip at three shear speeds: (D) $V_{s}=0$, (C) $V_{s}/U=3/4$, and (D) $V_{s}/U=3$. (**E**) Concentration profiles from the simulations shown in (B) to (D). Compared to the case of no shear, the curve for $V_{s}<U$ is only slightly perturbed, whereas for $V_{s}>U$, the tip is thinner and smoother. All simulations are conducted with interfluid diffusivity $D=1.21\times 10^{-10}\;m^{2}/s$ and Pe ≫ 10^3^. For the cases of $V_{s}=0$ and $V_{s}/U=3/4$, the interface was simulated at Pe = 4 × 10^4^. To measure the case of $V_{s}/U=3$, we lowered U, so Pe = 1 × 10^4^.

For the applied shear to alter C(r) appreciably, one would expect that the shear speed $V_{s}$ would need to be comparable to (or larger) than the interface speed, U. If it were much smaller, then it would not perturb the flow appreciably. At a fixed injection rate, because of the radial geometry of the cell, U∝1/r, so that U(r) eventually decreases below $V_{s}$. The data of Fig. 3C show that the thinning of the profile occurs abruptly at $r=r_{0}$. Therefore, in Fig. 4A, we compare the measured interface speed at $r=r_{0}$, $U\left(r=r_{0}\right)$, with $V_{s}$. The dashed line indicates where the two speeds are equal. The data are consistent with $r_{0}$ being determined by where the interface speed becomes comparable to $V_{s}$

To understand how the competition between $V_{s}$ and U affects the interface profile, we simulate the flow within the gap using COMSOL Multiphysics software. The specifics and parameters used are described in Materials and Methods. To be consistent with the experiments, we used an interfluid diffusivity of $D=1.21\times 10^{-10}\;{\text{mm}}^{2}/s$ that matches our experimental value. As in the experiments, we ensured Pe > 10^3^ in the simulation to reduce the effects of diffusion on the interface shape.

To isolate how the steady-state fluid interface depends on the relative shear and injection velocities, we use a rectilinear geometry to ensure that U is constant throughout each simulation. While the results are thus not directly comparable with experiments in a circular geometry where the interface velocity varies inversely with radius, they provide physical insight into the underlying smoothing mechanism.

The three profiles shown in Fig. 4 (B to D) show how shear affects the interface shape near its tip. Figure 4B shows the profile when there is no shear, i.e., $V_{s}=0$, while Fig. 4 (C and D) shows the profiles for $V_{s}/U=3/4$ and $V_{s}/U=3$, respectively. Figure 2E shows the concentration profiles, C(r), for these three cases. When $V_{s}<U$, the thickness of the intruding tongue of inner fluid is approximately the same as when there was no shear. When $V_{s}>U$, the tongue becomes appreciably narrower and has a more tapered tip. These simulations are consistent with our experiments and the argument that the smoothing occurs appreciably only when the applied shear is at least as large as the interfacial velocity.

In fig. S10, we also use these simulations to show how the inner fluid profile becomes established. The thickness of the tongue continues to decrease slightly during the first few oscillations of shear.

### Onset and bluntness correlation

Having identified $r_{0}$ as the characteristic radius where smoothing due to shear begins, we now examine how it relates to the onset of the fingering instability. There are two important experimental parameters, the normalized speed $V_{s}/Q$ and the amplitude $d_{m}$, that characterize the applied oscillatory shear. We show here that to affect the fingering onset, (i) the shear speed, $V_{s}/Q$, must be large enough that the smoothing occurs before the onset of finger growth, i.e., $r_{0}<R_{\text{on}}$, and (ii) the amplitude of shear, $d_{m}$, which alters the degree to which the profile becomes smoother, can likewise delay the instability only when $r_{0}<R_{\text{on}}$.

#### Role of shear speed

**Fig. 5.**
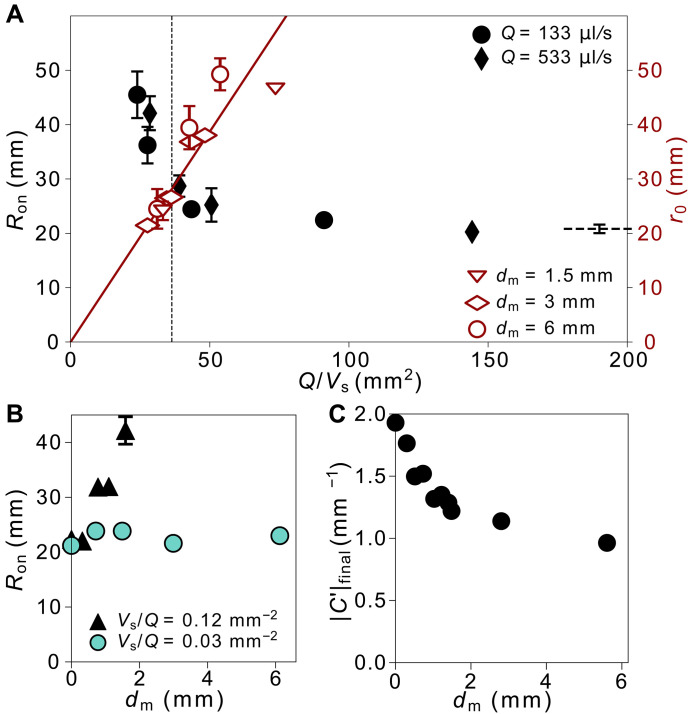
Competition between fingering onset, $R_{\text{on}}$, and smoothing radius, $r_{0}$, under shear. (**A**) $r_{0}\propto Q/V_{s}$ (red) while $R_{\text{on}}$ (black) decreases until the datasets cross at $R_{\text{on}}\approx r_{0}$ shown by vertical dashed line. To the left of their intersection, $r_{0}<R_{\text{on}}$ resulting in a significant delay in $R_{\text{on}}$. To the right, $r_{0}>R_{\text{on}}$, so the smoothing has only a mild influence on $R_{\text{on}}$. (Same $R_{\text{on}}$ dataset as in Fig. 2B.) (**B**) $R_{\text{on}}$ (black) increases significantly with shear amplitude, $d_{m}$, when $V_{s}/Q>0.027\;{\text{mm}}^{-2}$, the intersection value found in (A). For $V_{s}/Q<0.027\;{\text{mm}}^{-2}$, $R_{\text{on}}$ (teal) remains nearly independent of $d_{m}$. (**C**) ${|C^{\prime }|}_{\text{final}}$ decreases with $d_{m}$. For $d_{m}=1.5\;\text{mm},3\;\text{mm},\text{and}\;6\;\text{mm}$, ${|C^{\prime }|}_{\text{final}}$ is averaged over different values of $V_{s}/Q$ using the data in Fig. 6A. For all the other values of $d_{m}$, $V_{s}/Q=0.12\pm 0.01\;{\text{mm}}^{-2}$.

If the interface smoothing occurs after onset (i.e., $r_{0}>R_{\text{on}}$), there is no opportunity for shear to delay the onset and one would expect $R_{\text{on}}$ to remain nearly unchanged. This is corroborated by the data for $R_{on}$ and $r_{0}$ versus $Q/V_{s}$ in Fig. 5A. The plot shows $r_{0}\propto Q/V_{s}$, while $R_{\text{on}}$ does not vary markedly until the two datasets cross (i.e., $r_{0}\approx R_{\text{on}}$) shown by the vertical dashed line. Only to the left of this line, where the smoothing occurs before onset, does $R_{\text{on}}$ increase significantly.

#### Role of shear amplitude

The relative values of $R_{\text{on}}$ and $r_{0}$ also determines the effect of shear amplitude, $d_{m}$, on the onset radius $R_{\text{on}}$. As shown in Fig. 5B, at $V_{s}/Q=0.12\;{\text{mm}}^{-2}$ (black data), where $r_{0}<R_{\text{on}}$, increasing $d_{m}$ markedly delays $R_{\text{on}}$. Figure 5C shows that ${|C^{\prime }|}_{\text{final}}$ decreases with increasing $d_{m}$, indicating that the shear amplitude controls the final profile smoothness. As a result, the delayed onset with increasing $d_{m}$ at high shear speeds also correlates with a smoother profile. In contrast, when $V_{s}/Q$ decreases to $0.03\;{\text{mm}}^{-2}$, where $r_{0}>R_{\text{on}}$, increasing $d_{m}$ to 6 mm has a little effect on $R_{\text{on}}$. This corroborates that unless smoothing starts before onset, the shear amplitude, which controls how much smoothing occurs, does not affect the instability.

**Fig. 6.**
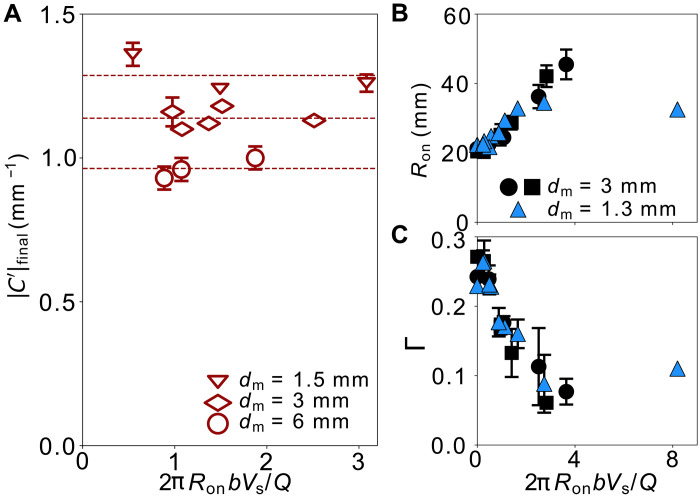
Role of shear to induced interface smoothness on instability suppression. (**A**) Final value of profile bluntness, ${|C^{\prime }|}_{\text{final}}$, is insensitive to dimensionless shear speed, $2\pi R_{\text{on}}{\mathrm{bV}}_{s}/Q$, for different shear amplitudes, $d_{m}$. At $d_{m}=1.3\;\text{mm}$, $R_{\text{on}}$ (**B**) and Γ (**C**) plateau at high $2\pi R_{\text{on}}{\mathrm{bV}}_{s}/Q$, showing a limit set by the corresponding ${|C^{\prime }|}_{\text{final}}$. In comparison, at $d_{m}=3\;\text{mm}$ (same data as in Fig. 2B), where ${|C^{\prime }|}_{\text{final}}$ is smaller than that of $d_{m}=1.3\;\text{mm}$, $R_{\text{on}}$ and Γ extend beyond the measurement limit.

Figure 6A shows that ${|C^{\prime }|}_{\text{final}}$ does not vary with the dimensionless shear speed, $2\pi R_{\text{on}}{\mathrm{bV}}_{s}/Q$, but only depends on $d_{m}$. Thus, once $r>r_{0}$, the profile remains at a constant smoothness even as $2\pi R_{\text{on}}{\mathrm{bV}}_{s}/Q$ increases; that is, ${|C^{\prime }|}_{\text{tip}}$ does not decrease below ${|C^{\prime }|}_{\text{final}}$, which depends only on the shear amplitude but not shear speed.

Consistent with the idea that the smoothness of the interface controls the onset of the fingering instability and the subsequent finger growth, this lower limit in the profile smoothness also sets the limiting value for both $R_{\text{on}}$ and Γ. This is seen in Fig. 6 (B and C) where at $d_{m}=1.3\;\text{mm}$, both $R_{\text{on}}$ and Γ reach a plateau for $2\pi R_{\text{on}}{\mathrm{bV}}_{s}/Q>2.7$ that is visible within our measurement window. As the shear amplitude increases to $d_{m}=3\;\text{mm}$, both $R_{\text{on}}$ and Γ continue to evolve until they exceed our measurement range.

## DISCUSSION

Previous experiments have indicated that a necessary condition for the formation of a viscous fingering instability in miscible fluids is the existence of a sufficiently blunt interface in the z direction spanning the gap (*9*–*13*), and here, we have directly addressed the role of the viscosity contrast in the case of low diffusion by applying oscillatory shear to perturb the three-dimensional interface profile. This work confirms the fundamental correlation between the smoothness of the gap profile at the edge of the pattern and the onset of the instability. It demonstrates the conditions under which shear produces a smoother interface and how the fingering onset radius and growth rate are influenced by the interface smoothness. There are two important shear variables, the shear speed and the shear amplitude, that play distinctly different roles.

### Different roles of shear speed, $V_{s}$, and shear amplitude, $d_{m}$

In order for shear to distort the inner fluid profile significantly, $V_{s}$ must be comparable to the velocity of the interface U(r) as shown in Fig. 4A. If $V_{s}$ were much smaller than this, then the dynamics would naturally be dominated by the injection rate, Q, rather than by the shear. Our data indicate that it is only in the regime $V_{s}>U\left(r\right)$ that the shear can effectively smooth the interface, C(r).

In a circular cell, as in our experiments, U(r)∝Q/r. This allows the condition $V_{s}=U\left(r\right)$ to occur at different radii. Initially, at small r, the interface moves rapidly compared to the shear speed, $V_{s}$, of the plates; at later times, after the interface has expanded, the interface speed drops below $V_{s}$. Thus, at large enough radius, the condition stated above for smoothing will always be met at some radius $r=r_{0}$. The competition between $V_{s}$ and U(r) explains why $r_{0}\propto \left(Q/V_{s}\right)$ in Fig. 5A. The speed of interface growth is $U\left(r=r_{0}\right)\approx Q/\left(2\pi {\mathrm{br}}_{0}\right)=V_{s}$, so that $r_{0}\approx Q/\left(2\pi {\mathrm{bV}}_{s}\right)$. For $r<r_{0}$, ${|C^{\prime }|}_{\text{tip}}$ remains constant and then drops abruptly (i.e., within our resolution) to ${|C^{\prime }|}_{\text{final}}$ at $r_{0}$ where the interface becomes smoother.

This alteration of the structure in the z direction changes the fingering instability onset and the subsequent growth rate. Crucially, our experiments demonstrate that the delayed onset can only occur when the effect of shear occurs early enough that it precedes the onset.

The shear amplitude, $d_{m}$, can only appreciably affect the inner fluid profile after the smoothing has started to occur, that is, after $r\geq r_{0}$. The interplay between $R_{\text{on}}$ and $r_{0}$ reveals a transition in the instability dynamics and growth rate, Γ. When $R_{\text{on}}\leq r_{0}$, the dynamics of the instability proceed as if there were no shear-induced thinning of the profile. In this region, the fingering dynamics are insensitive to $r_{0}$. This remains true even at high amplitudes, $d_{m}$. However, when $R_{\text{on}}>r_{0}$, the applied shear not only delays $R_{\text{on}}$ but also reduces the subsequent finger growth rate Γ. Consistent with the trend of $R_{\text{on}}$, Γ decreases with increasing $V_{s}$ and $d_{m}$.

One can revert to the no-shear limit by allowing either $d_{m}\to 0$ or $V_{s}\to 0$. As $d_{m}\to 0$, ${|C^{\prime }|}_{\text{final}}\to {|C^{\prime }|}_{\text{no}\;\text{shear}}$, meaning there is no reduction in bluntness and therefore no suppression of $R_{\text{on}}$ or Γ. On the other hand, as $V_{s}\to 0$, $r_{0}\to \infty$, so the interface never reaches the radius where ${|C^{\prime }|}_{\text{tip}}$ begins to drop. Hence, the smoothing effect cannot emerge in finite time, and no instability suppression occurs. Thus, while both the parameters $d_{m}$ and $V_{s}$ cause a delay in onset, they do so in different ways.

### Shear as a control parameter

This work introduces applied shear as a relevant perturbation that can be used to control viscous fingering. It offers practical advantages because it allows additional control over instability suppression independent of intrinsic fluid properties.

What is not intuitive to us is how the shape of the profile, C(r), gets established. This is observed in our experimental measurements of the concentration profiles, shown in Fig. 3 and corroborated in the COMSOL simulations, which are discussed in the Supplementary Materials. Initially, as the injection starts and the shear is applied, the shape of the profile near the tip varies markedly. After only a few cycles, it reaches a condition where the local profile returns to nearly the same shape on each oscillation. This is seen in Fig. 3C, where $C^{\prime }\left(r\right)$ reaches a constant value ${|C^{\prime }|}_{\text{final}}$. Thus, the interplay of the dynamics that creates the shape of the inner fluid tongue needs further study.

Other attempts to control the instability have been attempted. By lowering the injection rate, diffusion effects were increased to blur the concentration profile. While this initially delayed the instability, a different fingering instability, with a different wavelength and growth rate, emerged when the Péclet number was sufficiently low (*19*). In the Supplementary Materials, we discuss in detail why neither molecular diffusion nor scalar dispersion is the dominant mechanism responsible for the observed smoothing effect. Other studies have used nonuniform (*32*, *33*) or time-dependent (*3*) gap geometries, variable injection rates (*34*–*36*), deformable membranes (*37*–*39*), and electric fields (*40*, *41*) to modify the instability. Each of these methods has drawbacks and advantages. Applying mechanical shear offers a robust alternative, including the possibility of temporal control. Similarly, additive manufacturing and biomedical engineering could adopt shear-driven smoothing to enhance coating uniformity or vascular network fabrication.

## MATERIALS AND METHODS

### Experiments

The Hele-Shaw cell consisted of two circular confining glass plates with radii R=14 cm and a thickness greater than 1.27 cm to prevent bending. The gap spacing between the plates was b=305 μm and was kept uniform by six spacers located around the periphery of the plates. The fluids were injected using a syringe pump (NE-1000 from New Era Pump Systems Inc.) via a hole in the top plate of diameter 1.6 mm. The two plates were carefully aligned, and the outer fluid was injected to the edge of the cell. Before injecting the inner fluid, the outer fluid residue and bubbles inside the inlet were removed by flushing the less viscous displacing inner fluid through the injection tube to the inlet and pumping it out from the waste tube. The valve for the waste tube was then closed. The fluid viscosities were measured by an Anton Paar MCR 301 rheometer.

The bottom plate had no hole, so that during the applied shear, there would not be any perturbation to the flow caused by an opposing hole. This plate was leveled on top of three T-slotted framing rails with bumpers between the rails and plate. Screws were mounted on the rails to secure the bottom plate at rest. Bumpers were used between the screws and the bottom plate to reduce vibration. The top plate was enclosed by an aluminum ring connected to a 2-inch Feedback Rod Linear Actuator (FA-PO-35-12-2 from Firgelli Automations). An Arduino with a High Power Motor Drive (HiLetgo BTS7960 43A) was programmed to drive the actuator back and forth with a constant speed and amplitude.

Using two parallel lines, one on each plate, perpendicular to the direction of shear, the relative plate displacement was measured Fig. 1C. Considering the maximum displacements ($d_{m}$) were not always captured by the camera with a finite frame rate, we measure $d_{m}$ as half the difference between the two maximum excursions on each side centered around the beginning point over multiple cycles with an error of 0.06 mm. The shear speed $V_{s}$ was calculated as $4d_{m}/T_{s}$, where $T_{s}$ is the period averaged over multiple cycles. The error of $V_{s}$ is less than 3.6%.

The fingering patterns were imaged using a Prosilica GX 3300 camera from below the bottom plate. We plotted the interface between the two fluids in polar coordinates with θ=0 (θ=π) along the positive (negative) x axis. This is the axis of the shear. To determine the onset radii in different angular directions, we divide the interface into 32 equal segments with the center of the first segment at θ=0. The onset radius, $R_{\text{on}}$, is measured inside each segment. The valleys between fingers are sharper than the fingers themselves; we therefore measure the position of the first minimum with an amplitude of at least 0.2 mm from its nearest local maximum. The radial coordinate of this first detected minimum in the original interface segment is used as the onset radius for that segment. To reduce the influence of noise, we fit $R_{\text{on}}$ versus θ with a second-order Fourier component. We used the fitting results as the onset in the parallel directions.

At injection rate Q=67 μl/s, we tracked the interface until it reached r=50 mm, so U=Q/(2πbr)>0.7 mm/s. At larger injection rates, Q≥133 μl/s, we tracked the interface until it reached r=70 mm, so U=Q/(2πbr)>1 mm/s. We only measured $R_{\text{on}}$ within the radius that we tracked. As a result, for all our experiments, the Péclet number Pe≡Ub/D=Q/(2πrD)>1750.

To measure C(r), we dyed the inner fluid with 0.04 weight % of brilliant blue G-250 (Alfa Aesar) and calculated the concentration from calibrated intensity of transmitted light (*11*, *19*). The concentration profile C(r) is tracked along the direction from the center of the pattern to the tip of the interface. After fingers are merged, the profile is taken across the finger peaks for individual fingers closest to the parallel directions (θ=0 and θ=π). Before fingering, the profile is taken along the same azimuthal directions as the earliest-detected finger in subsequent frames.

The finger length $R_{f}$ is tracked for individual fingers close to the parallel directions. $R_{f}$ is the difference between the radial coordinate of a local maximum and the average of the radial coordinates of its adjacent minima on the interface.

### Simulations

The simulations were done using COMSOL Creeping Flow (spf) and Transport of Concentrated Species (tcs) coupled by Reacting Flow (nirf) under Multiphysics (COMSOL version 6.2.0.278) (*42*). We used a two-dimensional rectangular geometry with gap b=0.305 mm and length L=35 mm. The top and bottom walls have no slip boundary conditions with no flux. The initial inner fluid concentration was set to an error function $1/2\left\{1-\text{erf}\left[\left(x-5\right)/\delta \right]\right\}$, where δ=8.77 μm is the initial diffusion width. The left wall is the inlet with fully developed flow as the boundary condition, and the right wall is the outlet with a static pressure. To ensure consistent velocity initial conditions, U is smoothly increased from 0 with a transition zone of 0.01 s and two continuous derivatives. The top wall was assigned a translational velocity with a square wave function with $V_{s}=12\;\text{mm}/s$ as the amplitude and $4d_{m}/V_{s}=1\;s$ as the period. The phase shift for the square wave function is 0.25 s. To avoid singularities, the wall motion is smoothed to have a continuous second derivative and smoothly increased from 0 mm/s. The viscosities for the inner and outer fluids were set to be $\eta_{\text{in}}=35\;\text{mPa}\cdot s$ and $\eta_{\text{out}}=218\;\text{mPa}\cdot s$. The diffusion between the fluids was simulated using Fick’s law with diffusion coefficient $D=1.21\times 10^{-10}\;m^{2}/s$ (*19*). We used a mapped mesh with a rectangular shape as the initial mesh and regular adaptive mesh refinement with a rough global minimum around the inner-outer fluid interface to enhance resolution.

## Supplementary Materials

### This PDF file includes:

Supplementary Text Figs. S1 to S10 References
