# Supplementary material for: Effect of translational shear on interfacial structure in the viscous fingering instability
Source: Sci Adv. 2026 Apr 3;12(14):eaeb2907. doi: 10.1126/sciadv.aeb2907 (PMC13048239; doi:10.1126/sciadv.aeb2907)
Supplement: Supplementary file 1 — Supplementary Text Figs. S1 to S10 References [file sciadv.aeb2907_sm.pdf]

Science Advances
AAAS

Supplementary Materials for

Effect of translational shear on interfacial structure in the viscous fingering instability

Zhaoning Liu *et al.*

Corresponding author: Zhaoning Liu, znliu@uchicago.edu

Sci. Adv. **12**, eaeb2907 (2026)
DOI: 10.1126/sciadv.aeb2907

This PDF file includes:

Supplementary Text
Figs. S1 to S10
References

Role of Interface Bluntness on Onset

The shape of the interface as a less-viscous miscible fluid invades a more viscous one is determined by several distinct mechanisms. Aside from the role of shear, as discussed in the present paper, the viscosity ratio of the two fluids, η_{in}/η_{out}, plays a crucial role. As η_{in}/η_{out} increases towards unity, the profile of the inner fluid near its tip becomes smoother and R_{on} increases (*11*). That is, R_{on} increases with decreasing $|C'|_{tip}$ which is either due to an increasing η_{in}/η_{out} or to the presence of shear.

Here we compare how these two ways of smoothing the profile (*i.e.*, decreasing $|C'|_{tip}$) affect the onset. We vary $|C'|_{tip}$ by (i) varying the viscosity ratio without any shear or (ii) keeping the viscosity ratio constant and varying the shear amplitude. In both sets of experiments, we use identical Hele-Shaw cell geometries.

In the case that shear is applied, $|C'|_{tip}$ depends only on d_m, but R_{on} depends on both d_m and V_s, in a way that increasing V_s/Q decreases r_0 delaying R_{on} (Fig. 5A). However, as shown in Fig. 6B, when V_s/Q keeps increasing, $r_0 \to 0$ and R_{on} plateaus. For a sufficiently high shear speed (*e.g.*, $V_s/Q > 0.11$ mm^{-2}), the measured R_{on} is the one corresponding to $|C'|_{tip} = |C'|_{final}$. The shear amplitudes d_m vary between 0.3 mm and 1.6 mm.

We plot R_{on} versus $|C'|_{tip}$ for both sets of experiments in Fig. S1. The data share a similar trend and are close to each other, as shown in Fig. S1A. We note that if we scale the abscissa by a factor $(\eta_{out}/\eta_{in} - 1)$, the collapse of the two data sets is slightly better, as shown in Fig. S1B. More data is needed to establish which of these two scaling forms is a better fit.

Role of diffusion and scalar dispersion

Our analysis indicates that the smoothing of the concentration profile and the associated delay of the viscous fingering instability are not primarily caused by molecular diffusion or shear-induced scalar dispersion.

Molecular diffusion

Previous work showed that when the Péclet number is above 1000, the onset radius does not change with Pe, while when the Péclet number decreases below 1000, diffusion matters and the instability

enters a different regime where the R_{on} changes as Pe increases (*19*). In our experiments, we ensured Pe > 1750 even beyond R_{on}, so that Pe itself does not change the instability. If diffusion dominates, one would expect the concentration profile to be smoother as we lower the injection rate, Q. However, as shown in Fig. S2, both the concentration profile $C(r)$ and its derivative $C'(r)$ are insensitive to the injection rate over an order of magnitude, indicating that diffusion does not control the interfacial structure in the parameter space explored by our experiments.

A comparison of characteristic timescales further supports that diffusion does not dominate. The characteristic time for shear to penetrate across the gap, $\tau_{\text{gap}} = b/V_{\text{s}}$, ranges between 0.01s and 0.22s and the oscillation period, $\tau_{\text{osc}} = 4d_{\text{m}}/V_{\text{s}}$, ranges between 0.6s and 10s. In comparison, considering the diffusivity $D = 1.21\times10^{-10}\text{mm}^2/\text{s}$, the time for diffusion to relax the concentration profile across the gap is $\tau_{\text{diff}} \approx b^2/D = 768\text{s}$, which is at least 3000 times larger than τ_{gap} and 70 times longer than τ_{osc}. The interface evolution in our experiments typically takes within 120s, so diffusion cannot appreciably modify the concentration profile over the timescale for shear to suppress the finger growth.

Scalar dispersion

Shear-induced scalar dispersion is also suppressed by the oscillatory nature of the applied shear. The scalar dispersion can be derived from the advection–diffusion equation: $\partial C/\partial t = -\mathbf{v}_{\text{s}}\nabla c + D\nabla^2 c$, where $\mathbf{v}_{\text{s}}$ is the shear velocity, D is diffusivity, and c is the concentration. When $\mathbf{v}_{\text{s}}$ reverses direction from V_{s} to $-V_{\text{s}}$, it partially reverses the dispersion. We know from the literature that the dispersion decreases with increasing frequency when applying sinusoidal periodic $\mathbf{v}_{\text{s}}$ (*43*).To verify that a square-wave periodic shear velocity, as used in our experiments, also reduces scalar dispersion, we simulated a droplet with parabolic lateral boundaries confined between two parallel plates. The top plate was driven by translational oscillatory shear identical to that used in the experiments, with $V_{\text{s}} = 12$ mm/s. As shown in Fig. S3, compared with the single-direction shear, oscillation significantly reduces scalar dispersion. The gap profile approaches the one without shear as shear frequency increases.

If scalar dispersion matters in the frequency range we used in our experiments, one would expect a lower shear frequency to reduce the derivative of the concentration profile. However, as shown in Fig. S4, both $C(r)$ and $C'(r)$ do not vary with V_{s} at fixed d_{m}, suggesting that scalar dispersion

does not significantly smooth the interface in these experiments.

Early-time finger growth rate

At a constant injection rate, the interface propagation speed U decreases as the pattern size increases, which in turn reduces dR_f/dt. To account for this effect, we evaluate the finger growth rate relative to the interface growth rate by examining the ratio $\frac{dR_\mathrm{f}/dt}{dR_\mathrm{tip}/dt} = dR_\mathrm{f}/dR_\mathrm{tip}$. We know that when there is no shear, finger length initially grows exponentially with pattern size and then transitions to a linear regime, where $\Gamma = dR_\mathrm{f}/dR_\mathrm{tip}$ is a constant (*12*). This linear growth behavior persists under shear across different shear speeds, as shown in Fig. S5. We measure the finger growth rate by fitting the linear regime of each curve and using the slope of the fit as the value of Γ.

Concentration profile without shear

In Fig. S6A, we plot the evolution of the concentration profiles without shear for a direct comparison with the profiles when there is shear shown in Fig. 3A. Both experiments use the same Hele-Shaw cell geometry, parameters, and fluids. In the absence of shear, the interface tip remains blunt as the inner fluid expands outwards. This is shown in Fig. 3 and more obviously in Fig. S6B, where $|C'|_\mathrm{tip}$ only fluctuates but does not exhibit a sustained decrease with increasing radius as the inner fluid expands outwards.

Bluntness under shear

Figure. S7 shows more examples of how the interface tip bluntness $|C'|_\mathrm{tip}$ involves under different shear speeds V_s and shear amplitudes d_m. All of the examples can be fit with the step function as the case in Fig. 3C. Consistent with what we discussed in the Results section, at fixed d_m, higher shear speed V_s causes an earlier abrupt drop in bluntness, while it does not significantly influence the final plateau. In contrast, increasing d_m shifts the plateau downward.

Onset radius collapse before plateau for different shear amplitudes

As a supplement to Figure 2, We include more data for different shear amplitudes in Fig. S5. At large shear amplitudes $d_{\mathrm{m}} = 3$ mm, 4.5 mm, and 6 mm, the trend of onset radius versus dimensionless shear speed collapses into a single curve.

COMSOL simulations

We use COMSOL simulations to investigate how shear perturbs the shape of the interface during the first shear cycle, as shown in Fig. S9. After the top plate moves to the right during the first quarter of a cycle, the interface is dragged to be significantly tilted (Fig. S9A). When the top plate reverses direction to the left, the interface becomes sharper again (Fig. S9B,C), but still smoother and narrower compared with the no shear case (Fig. S9F,G). To investigate the long-time stability of the interface, we simulate the profile at the end of subsequent cycles (Fig. S10A-D). After multiple cycles, the interface becomes slightly thinner as shown in Fig. S10E, but the interface retains the overall shape of Fig. S9D.

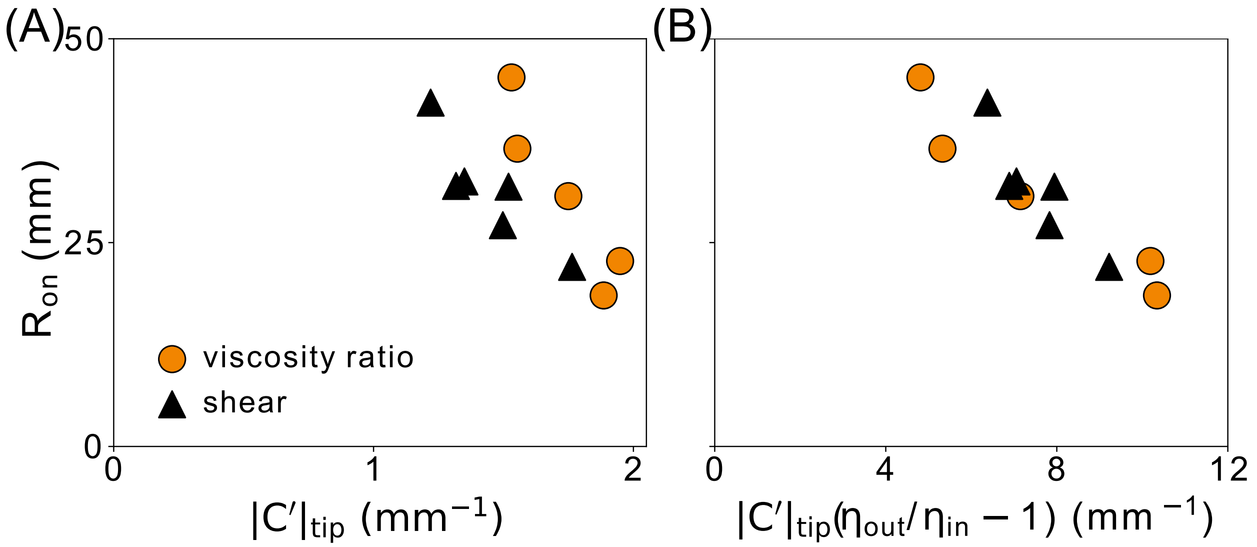

Figure S1: **Comparison of onset radius versus profile shape for two distinct control parameters. (A)** Onset radius, R_{on}, versus bluntness of interface tip, $|C'|_{\text{tip}}$. Black triangles: at fixed viscosity ratio ($\eta_{\text{in}}/\eta_{\text{out}} = 0.16$ as in manuscript) varying shear ($0.3\,\text{mm} < d_{\text{m}} < 1.6\,\text{mm}$) with $V_{\text{s}}/Q > 0.11\,\text{mm}^{-2}$. Oranges circles: no shear but varying viscosity ratio ($0.15 < \eta_{\text{in}}/\eta_{\text{out}} < 0.24$). Both data sets have the same overall trend. **(B)** Including a scaling factor of ($\eta_{\text{out}}/\eta_{\text{in}} - 1$) on the abscissa produces a somewhat better collapse of the two data sets; however, additional data are required to identify a convincing scaling factor.

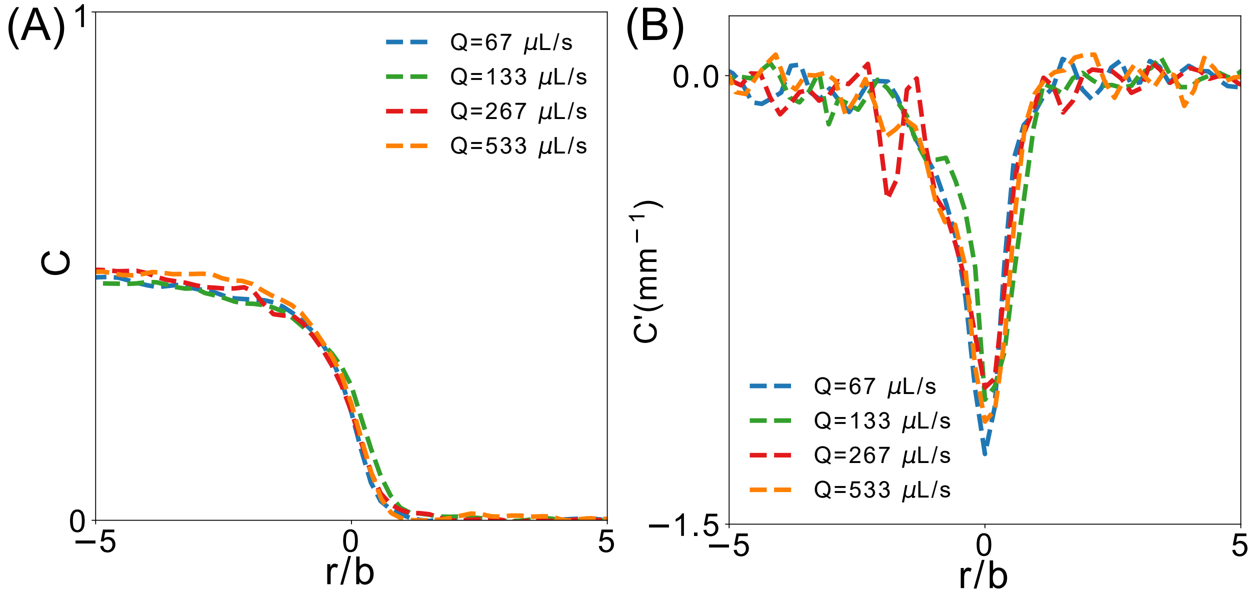

Figure S2: **The concentration profile $C(r)$ and its radial derivative $C'(r)$ for different injection rates Q.** Both $C(r)$ and $C'(r)$ are insensitive to the injection rates within experimental uncertainty. All data were taken at $d_\mathrm{m} = 3$ mm beyond the smoothing radius r_0.

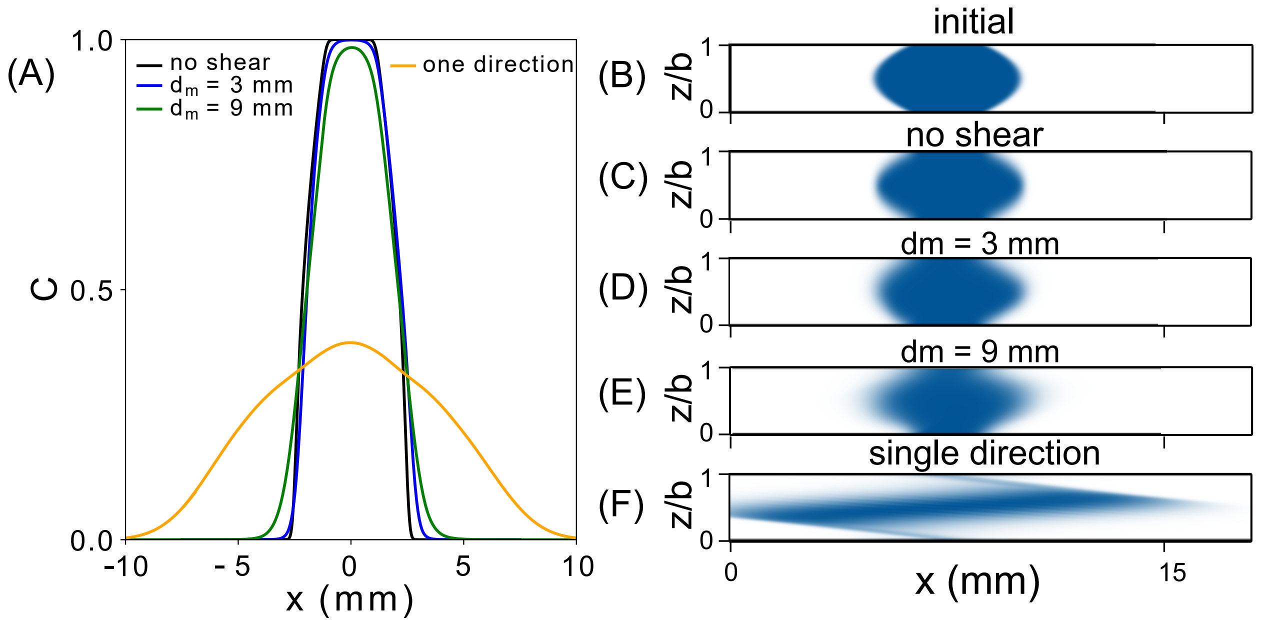

Figure S3: **COMSOL simulation of a stationary droplet under shear. (A)** Concentration profiles $C(r)$ for the case of no shear (black), d_m = 3 mm (blue), d_m = 9 mm (green), single-direction shear (orange) after 3 s. **(B)** Initial interface shape spanning the gap for the stationary droplet, with the interfluid interface governed by $(1 + \mathrm{erf}((x - 10 + 60(z - b/2)^2)/\delta))/2 + (1 - \mathrm{erf}((x - 5 - 60(z - b/2)^2)/\delta))/2$, where $\delta = 0.157$ mm. The length of the droplet on $z = b/2$ is 5mm. **(C-F)** The interface shape for the case of no shear **(C)**, d_m = 3 mm **(D)**, d_m = 9 mm **(E)**, and single-direction shear **(F)** after 3s. For the case of single-direction shear, we converted the coordinate back to rectangular using $x' = x - V_s t y/b$. For cases with shear, V_s = 12 mm/s.

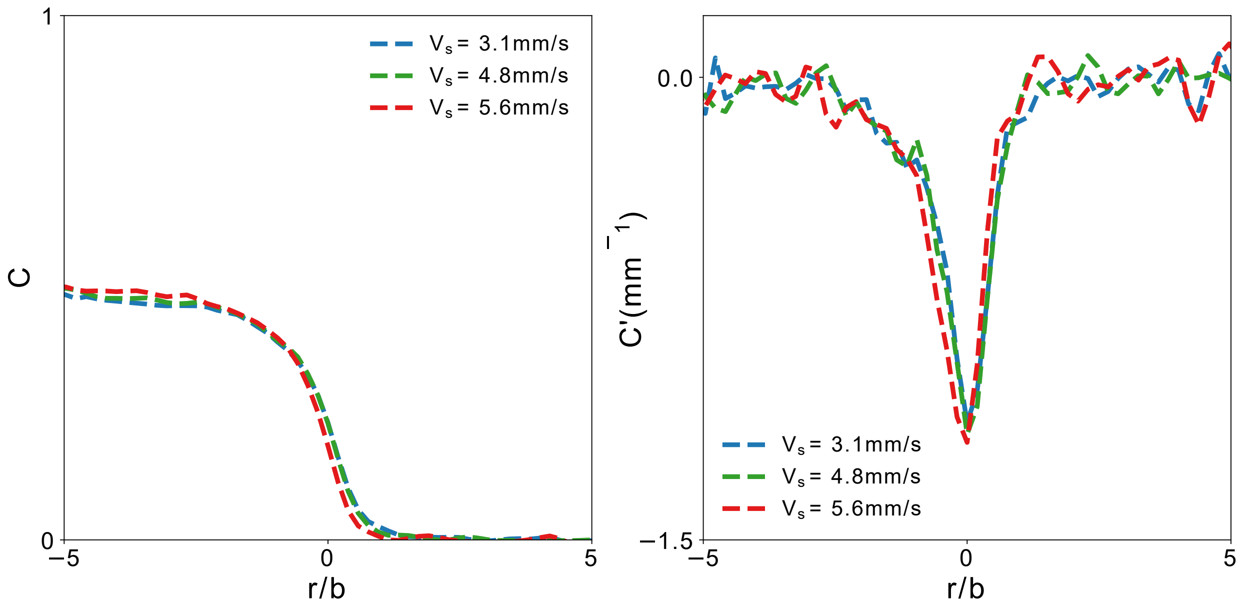

Figure S4: **The concentration profile $C(r)$ and its radial derivative $C'(r)$ for different shear speeds V_s at fixed d_m.** Both $C(r)$ and $C'(r)$ are insensitive to V_s, or frequency as d_m is fixed, within experimental uncertainty. All data were taken at $d_m = 3$ mm beyond r_0 where the interface tips were smoothed. $Q = 133\mu L/s$ for all data.

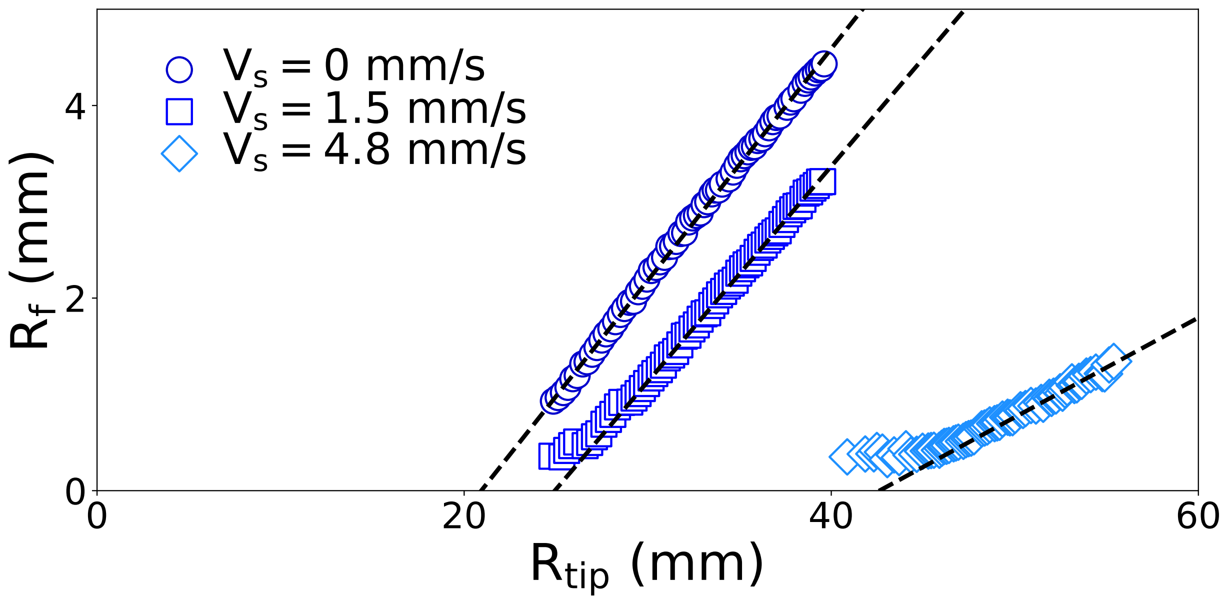

Figure S5: **Finger length** R_f **versus the pattern size** R_{tip}**.** For each dataset, the finger closest to the $\theta = \pi$ direction is tracked after R_f exceeds 0.2 mm. Following an initial exponential region, R_f grows linearly with R_{tip}, which can be fitted linearly with the dashed lines. The slope of each fit represents the finger growth rate $\Gamma = dR_f/dR_{tip}$, with steeper slopes corresponding to faster growth. For data with shear, $d_m = 3$ mm and $Q = 133$ μL/s.

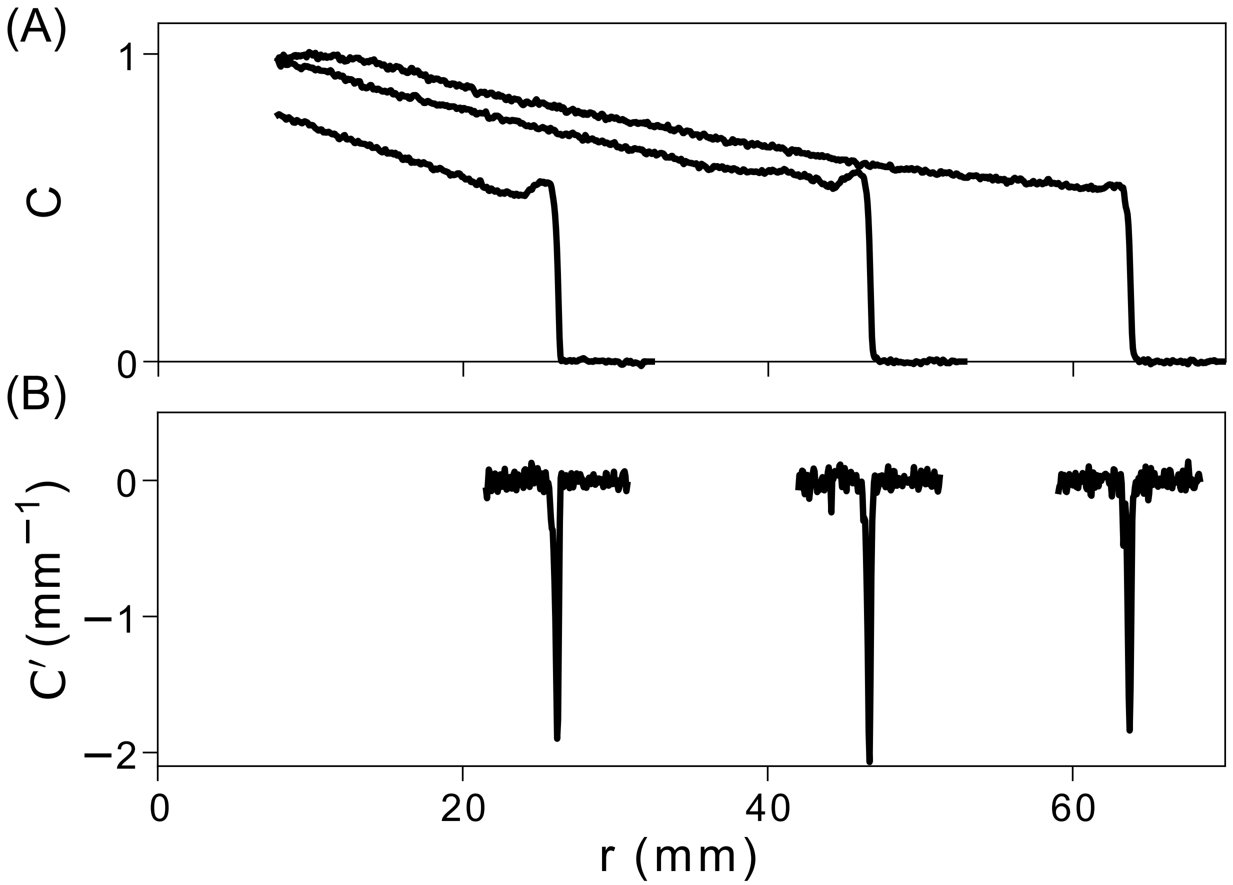

Figure S6: **Comparison of concentration profiles with and without shear (A)** Evolution of inner fluid concentration profiles $C(r)$ from an experiment without shear as the interface propagates outwards. The parameters for the Hele-Shaw cell geometry and the injection rate are the same as the case in Fig. 3A with shear. The curves from left to right correspond to the profiles at $R_{\text{tip}} \approx 26$ mm, 47 mm, and 64 mm. **(B)** Radial derivatives of concentration profiles $C'(r)$ near the tip, corresponding to the curves in **(A)**.

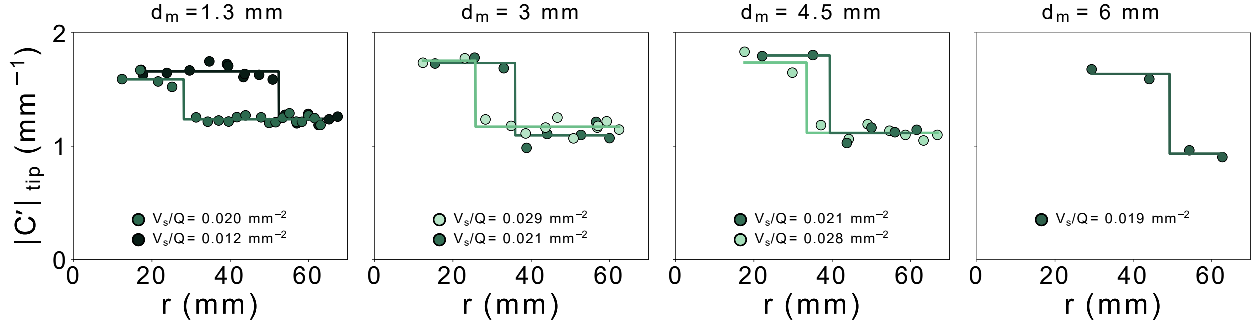

Figure S7: **Interface tip bluntness across different shear speeds and amplitudes**. Panels (left to right) correspond to shear amplitudes d_m = 1.3 mm, 3 mm, 4.5 mm, 6 mm. In all cases, a fit by a step function adequately fits the data to within our resolution.

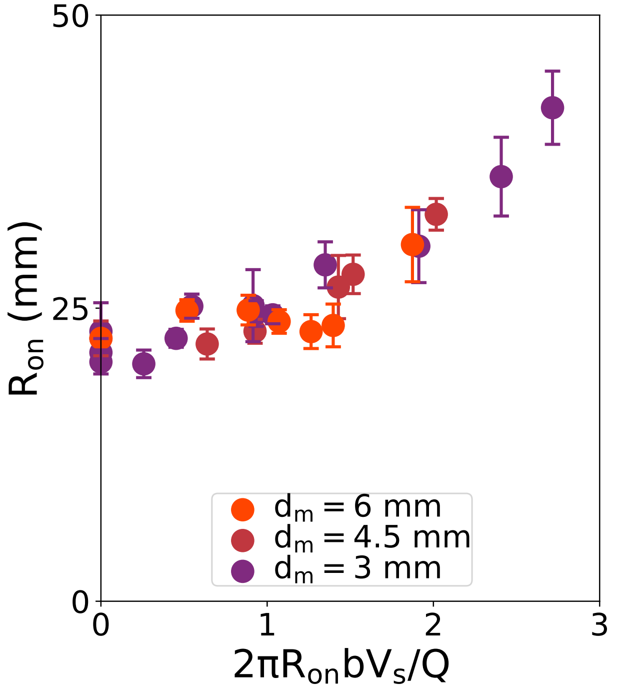

Figure S8: **Onset radius collapse before plateau for different shear amplitudes**. The shear amplitudes shown here are d_m = 3 mm (purple), 4.5 mm (deep red), 6 mm (light red).

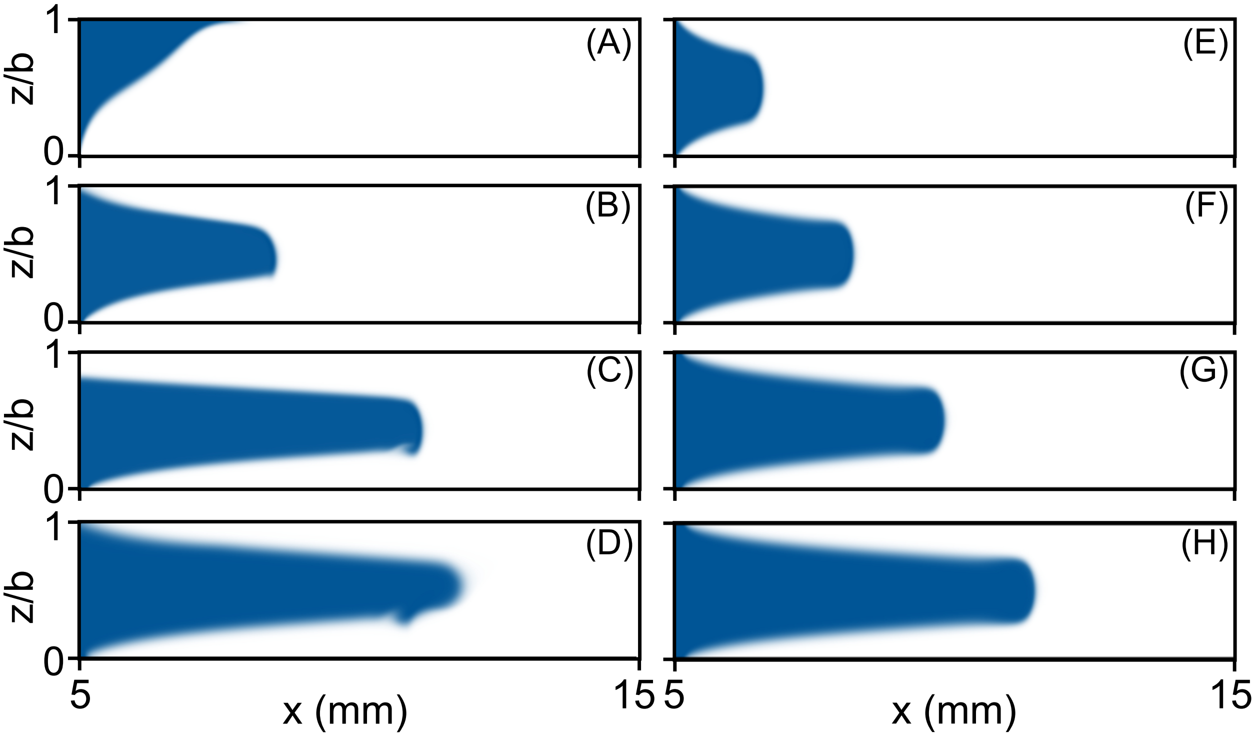

Figure S9: **Simulation showing shape of inner fluid during the first shear cycle**. **(A-D)** COMSOL simulation with V_s = 12 mm/s, U = 4 mm/s, and d_m = 3 mm during the first shear cycle **(A)** when the top plate reaches the right-most side. **(B)** when the top plate returns to the center after half a cycle. **(C)** when the top plate reaches the leftmost side. **(D)** when the top plate returns to the center after one cycle. **(E-H)** Simulations without shear at the same time as **(A-D)** respectively, using the same fluid properties, geometry, and interface velocity as the case with shear. Even when the plate is moving in the opposite direction to the interface, the tip under shear is still smoother and narrower. Simulations are done with the inner fluid initially filling the channel until 5 mm. The inner fluid is blue and the outer fluid is white.

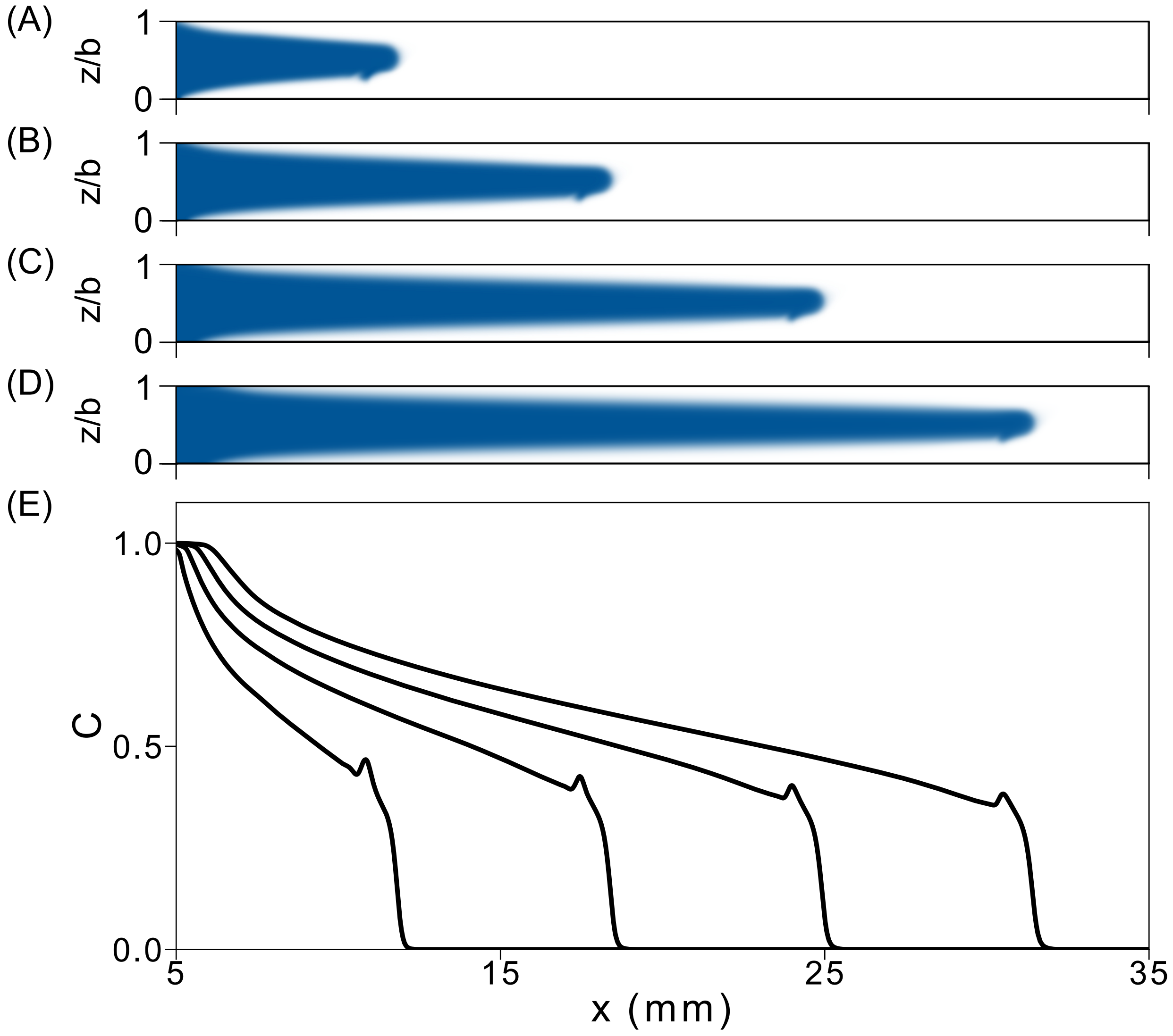

Figure S10: **Simulation of the inner fluid interface after multiple cycles. (A-D)** COMSOL simulation shows the shape of the inner fluid after one (A), two (B), three (C), and four (D) cycles. **(E)** The concentration profiles corresponding to the interfaces in (A-D) from left to right. The overall shape of the profile remains the same, although the width of the profile becomes slightly more slender at later times.

REFERENCES

1. P. G. Saffman, G. I. Taylor, The penetration of a fluid into a porous medium or Hele-Shaw cell containing a more viscous liquid. *Proc. A* **245**, 312–329 (1958).

2. D. Bensimon, L. P. Kadanoff, S. Liang, B. I. Shraiman, C. Tang, Viscous flows in two dimensions. *Rev. Mod. Phys.* **58**, 977–999 (1986).

3. Z. Zheng, H. Kim, H. A. Stone, Controlling viscous fingering using time-dependent strategies. *Phys. Rev. Lett.* **115**, 174501 (2015).

4. L. C. Morrow, C. Cuttle, C. W. MacMinn, Gas compression systematically delays the onset of viscous fingering. *Phys. Rev. Lett.* **131**, 224002 (2023).

5. E. Pitts, Penetration of fluid into a Hele–Shaw cell: The Saffman–Taylor experiment. *J. Fluid Mech.* **97**, 53–64 (1980).

6. R. A. Wooding, Growth of fingers at an unstable diffusing interface in a porous medium or Hele-Shaw cell. *J. Fluid Mech.* **39**, 477–495 (1969).

7. L. Paterson, Fingering with miscible fluids in a Hele Shaw cell. *Phys. of Fluids* **28**, 26–30 (1985).

8. M. Nagel, F. Gallaire, A new prediction of wavelength selection in radial viscous fingering involving normal and tangential stresses. *Phys. Fluids* **25**, 124107 (2013).

9. E. Lajeunesse, J. Martin, N. Rakotomalala, D. Salin, 3D instability of miscible displacements in a Hele-Shaw cell. *Phys. Rev. Lett.* **79**, 5254–5257 (1997).

10. E. Lajeunesse, J. Martin, N. Rakotomalala, D. Salin, Y. C. Yortsos, Miscible displacement in a Hele-Shaw cell at high rates. *J. Fluid Mech.* **398**, 299–319 (1999).

11. I. Bischofberger, R. Ramachandran, S. R. Nagel, Fingering versus stability in the limit of zero interfacial tension. *Nat. Commun.* **5**, 5265 (2014).

12. T. E. Videbæk, Delayed onset and the transition to late time growth in viscous fingering. *Phys. Rev. Fluids* **5**, 123901 (2020).

13. J. R. Lister, T.-F. Dauck, Fingering instability of self-similar radial flow of miscible fluids in a Hele-Shaw cell. *J. Fluid Mech.* **1008**, A31 (2025).

14. E. Lajeunesse, J. Martin, N. Rakotomalala, D. Salin, Y. Yortsos, The threshold of the instability in miscible displacements in a Hele–Shaw cell at high rates. *Phys. Fluids* **13**, 799–801 (2001).

15. B. Jha, L. Cueto-Felgueroso, R. Juanes, Fluid Mixing from Viscous Fingering. *Phys. Rev. Lett.* **106**, 194502 (2011).

16. B. Jha, L. Cueto-Felgueroso, R. Juanes, Quantifying mixing in viscously unstable porous media flows. *Phys. Rev. E* **84**, 066312 (2011).

17. C.-Y. Chen, E. Meiburg, Miscible displacements in capillary tubes. Part 2. Numerical simulations. *J. Fluid Mech.* **326**, 57–90 (1996).

18. C.-W. Park, G. Homsy, Two-phase displacement in Hele Shaw cells: theory. *J. Fluid Mech.* **139**, 291–308 (1984).

19. T. E. Videbæk, S. R. Nagel, Diffusion-driven transition between two regimes of viscous fingering. *Phys. Rev. Fluids* **4**, 033902 (2019).

20. P. Saffman, Viscous fingering in Hele-Shaw cells. *J. Fluid Mech.* **173**, 73–94 (1986).

21. F. Xu, J. Kim, S. Lee, Particle-induced viscous fingering. *J. Non-Newtonian Fluid Mech.* **238**, 92–99 (2016).

22. Y. Qiu, K. Xu, A. A. Pahlavan, R. Juanes, Wetting transition and fluid trapping in a microfluidic fracture. *Proc. Natl. Acad. Sci. U.S.A.* **120**, e2303515120 (2023).

23. C. Cuttle, L. C. Morrow, C. W. MacMinn, Compression-driven viscous fingering in a radial Hele-Shaw cell. *Phys. Rev. Fluids* **8**, 113904 (2023).

24. D. Li, Z. Yang, A. A. Pahlavan, R. Zhang, R. Hu, Y. F. Chen, Stability transition in gap expansion-driven interfacial flow. *Phys. Rev. Lett.* **133**, 034003 (2024).

25. L. V. McIntire, C. H. Lin, Finite amplitude instability of second-order fluids in plane Poiseuille flow. *J. Fluid Mech.* **52**, 273–285 (1972).

26. L. Talon, E. Meiburg, Plane Poiseuille flow of miscible layers with different viscosities: Instabilities in the Stokes flow regime. *J. Fluid Mech.* **686**, 484–506 (2011).

27. R. M. Oliveira, E. Meiburg, Miscible displacements in Hele-Shaw cells: Three-dimensional Navier–Stokes simulations. *J. Fluid Mech.* **687**, 431–460 (2011).

28. N. Goyal, E. Meibur, Miscible displacements in Hele-Shaw cells: Two-dimensional base states and their linear stability. *J. Fluid Mech.* **558**, 329–355 (2006).

29. N. Rakotomalala, D. Salin, P. Watzky, Miscible displacement between two parallel plates: BGK lattice gas simulations. *J. Fluid Mech.* **338**, 277–297 (1997).

30. I. Bischofberger, R. Ramachandran, S. R. Nagel, An island of stability in a sea of fingers: Emergent global features of the viscous-flow instability. *Soft Matter* **11**, 7428–7432 (2015).

31. S. D. Gowen, T. E. Videbæk, S. R. Nagel, Measurement of pressure gradients near the interface in the viscous fingering instability. *Phys. Rev. FLuids* **9**, 053902 (2024).

32. T. T. Al-Housseiny, P. A. Tsai, H. A. Stone, Control of interfacial instabilities using flow geometry. *Nat. Phys.* **8**, 747–750 (2012).

33. G. Bongrand, P. A. Tsai, Manipulation of viscous fingering in a radially tapered cell geometry. *Phys. Rev. E* **97**, 061101 (2018).

34. S. Li, J. S. Lowengrub, J. Fontana, P. Palffy-Muhoray, Control of viscous fingering patterns in a radial Hele-Shaw cell. *Phys. Rev. Lett.* **102**, 174501 (2009).

35. J. Y. Y. Chui, P. de Anna, R. Juanes, Interface evolution during radial miscible viscous fingering. *Phys. Rev. E* **92**, 041003 (2015).

36. J. Lawless, A. L. Hazel, A. Juel, On the oscillatory dynamics of a Saffman–Taylor finger with a bubble at its tip. *J. Fluid Mech.* **1020**, A39 (2025).

37. D. Pihler-Puzović, P. Illien, M. Heil, A. Juel, Suppression of complex fingerlike patterns at the interface between air and a viscous fluid by elastic membranes. *Phys. Rev. Lett.* **108**, 074502 (2012).

38. D. Pihler-Puzović, A. Juel, M. Heil, The interaction between viscous fingering and wrinkling in elastic-walled Hele-Shaw cells. *Phys. Fluids* **26**, 022102 (2014).

39. G. G. Peng, J. R. Lister, Viscous-fingering mechanisms under a peeling elastic sheet. *J. Fluid Mech.* **864**, 1177–1207 (2019).

40. T. Gao, M. Mirzadeh, P. Bai, K. M. Conforti, M. Z. Bazant, Active control of viscous fingering using electric fields. *Nat. Commun.* **10**, 4002 (2019).

41. M. Mirzadeh, M. Z. Bazant, Electrokinetic control of viscous fingering. *Phys. Rev. Lett.* **119**, 174501 (2017).

42. COMSOL Multiphysics v.6.2.0.278 (COMSOL); www.comsol.com (2023).

43. N. Poddar, D. Das, S. Dhar, K. K. Mondal, On scalar transport in an oscillatory Couette–Poiseuille flow under the effects of heterogeneous and bulk chemical reactions: A multi-scale approach. *Phys. Fluids* **35**, 043617 (2023).
